# Vitamin D in male sexual functions: unwrapping the sunshine hormone activities in erectile function and beyond

**DOI:** 10.3389/frph.2025.1594664

**Published:** 2025-08-22

**Authors:** Adeyemi Fatai Odetayo, Grace Edet Bassey, Grace Ayobami Fajemidagba, Kazeem Bidemi Okesina, Moses Agbomhere Hamed

**Affiliations:** ^1^ Department of Physiology, Faculty of Basic Medical Sciences, Federal University of Health Sciences, Ila-Orangun, Nigeria; ^2^ Department of Medical Physiology, Faculty of Basic Medical Sciences, University of Uyo, Uyo, Nigeria; ^3^ Department of Physiology, Redeemers University, Ede, Nigeria; ^4^ Department of Medical Physiology, School of Medicine and Pharmacy, College of Medicine and Health Sciences, University of Rwanda, Kigali, Rwanda; ^5^ Department of Medical Laboratory Science, Afe Babalola University, Ado-Ekiti, Nigeria; ^6^ The Brainwill Laboratories and Biomedical Services, Osogbo, Nigeria

**Keywords:** antioxidant, erectile dysfunction, endothelial function, penile erection, sexual dysfunction, vitamin D

## Abstract

**Background:**

Vitamin D, sometimes referred to as the “sunshine vitamin”, is well-known for its role in maintaining bone health processes that are highly dependent on calcium regulation. However, there is an emerging wealth of evidence that this fat-soluble vitamin has an important role in male sexual health regarding erectile function, production of testosterone, and overall fertility. Due to the increased vitamin D deficiency rates in most populations, the implications of its deficiency on male sexual functions have gained great interest.

**Materials and methods:**

This review, therefore, sums up recent publications that detail the association of vitamin D with male sexual functions regarding its potential roles in erectile function regulation, testosterone levels, and semen characteristics.

**Results:**

Vitamin D contributed positively to sexual and erectile functions. These were mediated via hormone-dependent mechanisms through its inhibitory role on prolactin and steroidogenic activities. It also improves endothelial functions by stimulating the secretion and release of nitric oxide, which is important for erection. Furthermore, it acts by mediating the activities of monoamine neurotransmitters, which are responsible for the motor activities involved in sexual function.

**Conclusion:**

Vitamin D acts via multiple mechanisms to enhance sexual and erectile functions.

## Graphical Abstract

**Figure:**
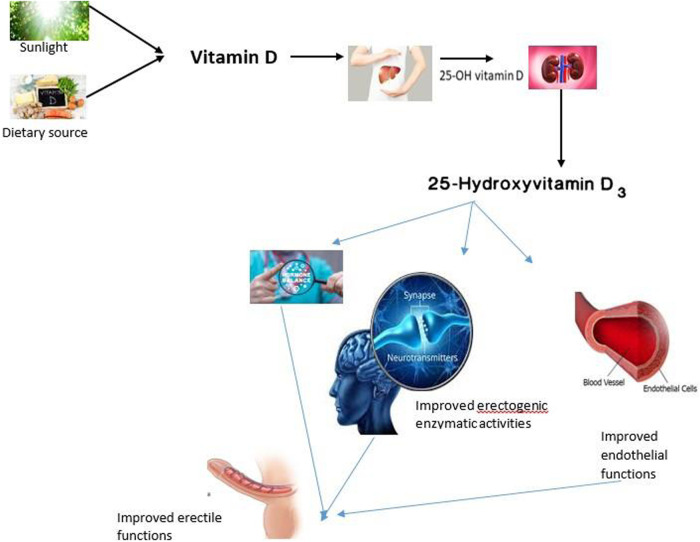

## 1 Introduction

Sexual function is an integral part of human health and is crucial for a good quality of life (1). Loss of sexual function is a major disorder, and it is mainly caused by erectile dysfunction (2). Although male sexual dysfunction can occur at any age, erectile function and libido decline with age (3). Erectile dysfunction is a disorder with an increasing rate of occurrence. As of 1995, about 150 million men were suffering from erectile dysfunction; meanwhile, about 322 million people were projected to likely have the disorder by 2025 (4). Erectile dysfunction is prevalent in the US, affecting 18–30 million men (5). Much of the reported upsurge could be attributed to a deteriorating diet and other unhealthy lifestyle choices (6). To support this, data from the National Health and Nutrition Examination Survey revealed that the population of US citizens with optimal serum vitamin D levels decreased by 49% between 1994 and 2004 (7), a period coinciding with an increase in the incidence of erectile dysfunction in the country.

Vitamin D is a micronutrient essential for optimal health throughout different stages of life. Vitamin D is a steroid hormone that can be obtained from the diet or synthesized in the human skin in the presence of sunlight, primarily the ultraviolet-B portion of sunlight, which accounts for approximately 80% of vitamin D (6). Recently, it has been established that vitamin D plays a more diverse role in humans than just maintaining calcium homeostasis (8, 13). The presence of vitamin D receptors (VDRs) in almost all human tissues, including those responsible for reproduction, indicates that vitamin D regulates numerous cellular differentiation and functions across different cell types (9–11). Vitamin D acts by binding to its receptor to stimulate genomic and non-genomic effects (12). After binding, the receptor translocates from the plasma membrane to the nucleus, bringing about gene activation via the hormone response element (13). According to the Human Genome Project, human DNA contains approximately 20,000–25,000 genes (14). Notably, approximately 3,000 out of these genes are responsive to vitamin D (13), further underscoring the numerous roles of vitamin D in human function. Therefore, if we consider that vitamin D has diverse functions in various tissues, vitamin D becomes more important for a healthy sexual life, especially for erectile function. Hence, this study reviewed existing literature to establish the sexual and erectile enhancing activities of vitamin D.

## 2 Vitamin D

Vitamin D is a secosteroid because of the ruptured B ring of the canonical steroid structure. It is produced from 7-dehydrocholesterol, a metabolite obtained during cholesterol synthesis, and can also be obtained directly from the diet. Vitamin D can be classified into vitamin D3 (cholecalciferol) or vitamin D2 (ergocalciferol). Cholecalciferol is produced in the skin (especially during summer months) after ultraviolet light or sunlight exposure. Vitamin D3 can also be obtained from animal-sourced foods, while ergocalciferol is obtained from plant sources and fortified foods. The major difference between the two forms of vitamin D is found in their side chain since vitamin D2 has a double bond between C22 and C23 and a methyl group at C24 in the side chain (15). However, these differences do not affect metabolism (16, 17). In fact, both forms of vitamin D have been established to have similar responses in the body (18). However, Tripkovic et al. (19) identified vitamin D3 as more effective at increasing serum 25(OH)D than vitamin D2; thus, vitamin D3 could be a preferred choice for supplementation.

The two forms of vitamin D are considered biologically inactive until they undergo two enzymatic hydroxylation reactions. The first enzymatic reaction occurs in the liver in the presence of 25-hydroxylase, especially the cytochrome P450 2R1, which forms 25-hydroxyvitamin D. The second hydroxylation reaction occurs in the kidney, and it is mediated by 1α-hydroxylase, where 25-hydroxylase is converted to the biologically active form, calcitriol or 1,25-dihydroxyvitamin D (20). Notably, the expression of the 1α-hydroxylase gene has been identified in various extra-renal tissues, although their roles have not been fully elucidated (21). However, the likelihood of this enzyme acting on vitamin D metabolites in these extra-renal organs as something other than the calcium-regulating hormone is more likely a contribution to circulatory 1,25-dihydroxyvitamin D in these tissues. 25-hydroxyvitamin D is the main circulating form of vitamin D (22), and it is found bound to a particular carrier protein known as vitamin D binding protein (23). This binding protein also helps in the transportation of vitamin D (24).

As previously stated, the biological activities of vitamin D involve gene expression regulation at the transcriptional level, which is mediated by binding to VDRs. These receptors are widely distributed throughout the body, even in organs that are not directly involved in maintaining calcium and phosphate homeostasis. The presence of these receptors in various organs suggests that vitamin D can perform multiple functions beyond calcium homeostasis (25). Additionally, the specific vitamin D-responsive elements are widely present in human genes involved in different activities such as cell differentiation and apoptosis (26). In fact, vitamin D has been suggested to have the specific vitamin D-responsive element activities via autocrine and paracrine pathways (27). Furthermore, Masuda and Jones (93) further hypothesized that vitamin D may have anti-cancer properties and can serve as a therapeutic agent for managing other chronic conditions, such as metabolic disorders.

### 2.1 The relationship between vitamin D and testosterone production

Testosterone is an important hormone; it plays a leading role in different physiological processes that are necessary to maintain normal male sexuality, such as libido, erectile function, and reproductive health overall (28). The main production site is in the testicles, and it has a major developmental effect on male secondary sexual characteristics, bone geometry, and lean muscle mass. Recently, much interest and research have been directed at the interrelationship between vitamin D levels and testosterone production.

The presence of VDRs in the male reproductive system suggests that vitamin D may play a crucial role in reproductive hormone synthesis (29). Therefore, understanding the relationship between vitamin D and testosterone levels will improve our knowledge of male reproductive health. Several studies have investigated the interplay between them, providing valuable insights into the relationship between vitamin D and testosterone.

According to Hu et al. (30), who restricted their work to men with type 2 diabetes, it was noted that those with hypogonadism had significantly lower levels of vitamin D compared to the normal group with testosterone. The findings from their cross-sectional data indicated a positive association between the level of serum 25(OH)D and total testosterone concentration. Another cross-sectional study involving 382 Malaysian men revealed a link between vitamin D, testosterone, and serum hormone-binding globulin (31). Also, according to a study by Wentz et al. (32) on 796 US male soldiers and veterans, a high vitamin D deficiency was observed and was associated with suppressed circulatory testosterone (32).

Similarly, Chen et al. (33) obtained from an MR analysis conducted on 4,254 Chinese men that a genetic decrease in 25-hydroxyvitamin D was associated with a decrease in testosterone levels. Additionally, Rafiq et al. (34) established a positive correlation between vitamin D and both total and bioavailable testosterone levels in a cross-sectional study conducted on 643 Dutch men. Strikingly, they also revealed that the positive correlation was not associated with sex hormone-binding globulin, estrogens, or gonadotropin levels, suggesting a specific influence on testosterone without altering the overall hormonal profile. Therefore, the fact that vitamin D is positively related to testosterone, without necessarily increasing the overall reproductive hormonal profile, suggests a direct effect on testicular functions rather than its effect on the hypothalamic-pituitary-gonadal axis.

The direct effect of vitamin D on the testis is supported by the study of Hong et al. (35), which revealed that vitamin D improved steroidogenesis by upregulating the expression of steroidogenic enzymes, including 17α-hydroxylase (CYP17A1), 17β-hydroxysteroid dehydrogenase (HSD17B1), and aromatase (CYP19A1). Similarly, Elrashidy et al. (36) observed that vitamin D improved the expression of steroidogenic acute regulatory protein (StAR), CYP11A1, 3β-hydroxysteroid dehydrogenase (HSD3b), and HSD17b in cisplatin-induced testicular dysfunction. These finding supports the hypothesis that vitamin D is an important factor in testosterone production, especially in populations with multiple health issues such as diabetes, in whom hormonal regulation commonly goes awry.

The positive influence of vitamin D on testicular steroidogenesis may be attributed to its ability to enhance testicular health by maintaining redox balance and preventing inflammation within the testicular tissue. Vitamin D supplementation can reduce nuclear factor kappa B (NF-κB) (37, 38) and nuclear factor erythroid 2-related factor 2 (Nrf2) (39, 40), which are the main regulators of inflammatory response and oxidative status (41). Additionally, vitamin D can directly enhance peroxisome proliferator-activated receptor gamma (PPAR-γ) expression (42), which can upregulate Nrf2 or even work synergistically with Nrf2 to maintain optimal redox balance and inhibit inflammation by suppressing NF-κB (43). Additionally, the interaction between PPAR-γ and vitamin D receptors, as well as the retinoid X receptor, also contributes to these protective effects against the oxidative-inflammatory response (42). The antioxidant property of vitamin D is important for improved testicular steroidogenesis, as studies have indicated that oxidative stress can lead to decreased testosterone levels. This suggests that vitamin D can be employed in the management and prevention of oxidative stress, which is crucial for maintaining healthy testosterone levels.

In contrast, Ulrich et al. (44) reported no significant changes in testosterone following vitamin D supplementation in both healthy individuals and those undergoing hemodialysis. This discrepancy, therefore, highlights the need for further research on the complex interplay between vitamin D and testosterone production. Such interactions could easily be altered depending on factors concerning age or comorbid states, in addition to basal vitamin D status, hence demanding a more blurred view or regard for the effect vitamin D has on testosterone levels among different populations.

### 2.2 The role of vitamin D in regulating sperm quality and quantities

There is growing interest in the link between vitamin D and male fertility, with numerous research articles highlighting its impact on sperm quality, motility, density, and morphology. Vitamin D plays several roles in the human body's systems, including the male reproductive system. Cito et al. (45) performed a systematic review and revealed that vitamin D receptors are present in various regions of the male reproductive system, with evidence suggesting that vitamin D may directly affect sperm parameters. This expression suggests that vitamin D may play a significant role in regulating male fertility, with a connection to sperm production and function.

In their review, there was an indication of a positive correlation between Vitamin D levels and sperm motility. Increased quality, in this case, is higher sperm motility, which helps with fertility since it determines how the sperm can reach the egg and fertilize it. However, the review also indicated inconsistencies in the findings of different research studies. The research showed that there is evidence of improved sperm motility in vitamin D deficiency, while other research found that vitamin D does not affect sperm motility. Such variability necessitates further specific experimental investigation to elucidate how vitamin D may influence serological characteristics.

Bank et al. (46) have once again examined the effects of vitamin D on couples with mild male factor infertility. In their study, they accounted that, vitamin D supplementation does not harm the sperm of the male and also has no influence on the major sperm production indicators, however, pregnant women whose male partners had low levels of vitamin D had increased pregnancy loss rates thus pointing to the possibility of vitamin D to have indirect impacts on fertility outcomes. This finding suggests that insufficient vitamin D may be detrimental to reproductive success, as reproductive challenges can still occur even when sperm values are normal. It is therefore vital that special emphasis be placed on the vitamin D status of men who are planning a family or are sexually active, as the implications of vitamin D deficiency, especially during pregnancy, are well-documented.

Additionally, Ciccone et al. (47) presented a significant association between serum vitamin D levels and various sperm parameters in men with semen abnormalities. It was observed that a high level of the vitamin increased the amount of sperm and motility; therefore, this study supported the hypothesis that it can promote male reproductive potential. These results contribute further to the existing literature on the positive effects of vitamin D on various fertility parameters in males. Additionally, Odetayo et al. (48) demonstrated that vitamin D increased sperm count, normal morphology, and motility in type 2 diabetes mellitus Wistar rats, and attributed their findings to the antioxidant and anti-inflammatory activities of vitamin D.

The ability of vitamin D to maintain spermatogenesis may be linked to its role in preserving the health and function of the blood-testis barrier (BTB), which is essential for proper spermatogenesis and male fertility. While the exact mechanisms are still being investigated, vitamin D's effects on the BTB are likely mediated through its interaction with VDRs and its influence on Sertoli cell function, as well as the regulation of the microenvironment within the seminiferous tubules. Vitamin D3 has been shown to bind with the active site of A Disintegrin and Metalloproteinase 17 (ADAM17) with a high binding score (49). ADAM17 has inhibitory effects on miR-145 expression (50). This miR-145 influences spermatogenesis by affecting the proliferation and differentiation of germ cells and Sertoli cells. Aberrant expression of miR-145 has been linked to male infertility, potentially affecting sperm quality and leading to conditions like azoospermia or oligospermia (51). Therefore, the ability of vitamin D to enhance ADAM17 can improve spermatogenesis by inhibiting miR-145 expression since minor changes in miRNA expression substantially affect the function of germ cells, such as impaired spermatogenesis and compromised fertility (52). Additionally, vitamin D has been shown to improve gamma-glutamyl transpeptidase activity, glucose uptake, LDH activity, and lactate production within the Sertoli cells, which are essential for the nutrition of germ cells and for ongoing, full, and active spermatogenesis (53). Therefore, vitamin D also maintains spermatogenesis by ensuring optimal metabolic activities within the testicular tissue, since glucose, lactate, and LDH are markers of energy balance (54).

### 2.3 Studies examining the correlation between vitamin D levels and fertility

Some recent papers have attempted to assess the relationship between vitamin D concentrations and fertility rates in males. For instance, observational studies by Adamczewska et al. (55) revealed that patients' vitamin D levels were correlated with sperm parameters; however, interventional studies showed no exact effect of vitamin D or its deficiency on fertility rates. These cross-sectional relations raise a question about male fertility outcomes and the potential mechanisms through which vitamin D may act; additional substantive work is needed to understand these relationships.

Maghsoumi-Norouzabad et al. (56) examined the tendency of sperm decrease by vitamin D supplementation to improve symptoms in infertile men with Asthenozoospermia. Although their study aimed to investigate the effect of vitamin D on sperm quality, the findings highlighted the need for future studies to establish the role of vitamin D in male fertility, particularly in relation to oxidative stress and hormonal responses. They postulated that, although Vitamin D influences sperm parameters, further research studies are required to provide definitive conclusions.

Amini et al. (73) obtained similar results and attributed self-identified vitamin D supplementation effects to changes in sperm characteristics, although sex hormone-binding globulin (SHBG) levels also changed. This implies that, whereas vitamin D does not enhance sperm concentration and motility, it may have an impact on other hormonal feedback mechanisms related to male fertility. However, a clear comprehension of mutual interactions between vitamin D and sex hormones is necessary for the generation of specific therapeutic approaches in cases of male infertility (Table 1).

**Table 1:** Summary of findings from previous studies on the role of vitamin D on male fertility.

Study	Study design	Sample size	Participant	Primary outcome
Odetayo et al. (57)	*In vivo* study	36	Wistar rats	Vitamin D enhances testicular function in type 2 diabetic rats
Chen et al. (33)	MR analysis	4,254	Chinese men	Genetic decrease in 25-hydroxyvitamin D linked to decreased testosterone levels
Rafiq et al. (34)	Cross-sectional	643	Dutch men	Positive correlation between vitamin D and testosterone levels
Chin et al. (31)	Cross-sectional	382	Malaysian men	Association between vitamin D and testosterone levels
Wentz et al. (32)	Cross-sectional	796	US men	Vitamin D deficiency linked to suppressed testosterone levels
Hu et al. (30)	Cross-sectional	N/A	Type 2 diabetes men	Positive association between vitamin D and testosterone levels
Hong et al. (35)	*In vitro*	N/A	Granulosa cells from porcine ovary of Pigs	Vitamin D improves steroidogenesis and testosterone production
Elrashidy et al. (36)	*In vivo*	50	Male Wistar Rats	Vitamin D improves steroidogenic enzymes and testosterone production
Ulrich et al. (44)	Randomised trial	N/A	Healthy & dialysis patients	No significant change in testosterone after vitamin D supplementation
Książek et al. (58)	Cross-sectional	N/A	Young, healthy men	No significant effect of vitamin D status on testosterone levels
Zittermann et al. (59)	Cross-sectional	N/A	Heart failure patients	Vitamin D supplementation couldn't stop testosterone decline in heart failure patients
Adamczewska et al. (55)	Systematic review	N/A	N/A	Positive relationship between vitamin D and testosterone and sperm parameters
D'Andrea et al. (60)	Meta-analysis	N/A	N/A	Inconsistency in vitamin D supplementation effects on testosterone levels
Lerchbaum et al. (61)	Randomised controlled trial	N/A	Low testosterone men	No significant effect of vitamin D supplementation on testosterone levels
Cito et al. (45)	Systematic review	N/A	N/A	Vitamin D receptors in male reproductive system; positive correlation with sperm motility
Banks et al. (46)	Observational study	N/A	Male infertility couples	No significant effect on sperm production but higher pregnancy loss rates in women with low partner vitamin D
Ciccone et al. (47)	Observational study	N/A	Men with semen abnormalities	Positive correlation between vitamin D and sperm count, motility, and fertility potential
Odetayo et al. (48)	Experimental study	30	Wistar rats	Vitamin D increased sperm count, morphology, and motility; linked to antioxidant and anti-inflammatory properties
Adamczewska et al. (55)	Observational study	N/A	N/A	Vitamin D levels correlated with sperm parameters; no definite effect on fertility rates
Maghsoumi-Norouzabad et al. (56)	Cross-sectional study	N/A	Infertile men with Asthenozoospermia	A need for future research on vitamin D's role in fertility, especially in oxidative stress
Amini et al. (73)	Observational study	N/A	N/A	Vitamin D supplementation affects sex hormones, but shows no significant impact on sperm concentration and motility
Yan et al. (62)	Meta-analysis	N/A	N/A	Low vitamin D levels are linked to decreased sperm concentration, motility, and morphology; emphasizes the need for adequate vitamin D in male fertility
Crafa et al. (63)	Meta-analysis (Cross-sectional studies)			Found that vitamin D deficiency is linked to severe erectile dysfunction (ED). Vitamin D deficiency worsened ED, independent of testicular function.
Demirci et al. (64)	RCT (Tadalafil + Vitamin D supplementation)			Vitamin D supplementation improved erectile function outcomes in men with vitamin D deficiency.
Farag et al. (65)	Cross-sectional study			Found lower vitamin D levels in men with ED compared to healthy subjects.
Tirabassi et al. (66)	Cross-sectional study			Concluded ED was linked to vitamin D deficiency, independent of testosterone levels.
Barassi et al., (67)	Cross-sectional study			Severe ED common in men with low vitamin D levels.
Caretta et al. (68)	Cross-sectional study			Identified vitamin D as a diagnostic marker for sexual dysfunction in diabetic patients.
Zhu et al. (69)	Animal model study			Found that vitamin D improved erectile function in cavernous nerve injury in rats
Fernandes-Lima et al. (70)	Animal model study			Concluded that vitamin D is required for maintaining penile cytoarchitecture.

In a recent review and meta-analysis by Yan et al. (62), the authors once again emphasized the relevance of vitamin D levels to male fertility. Their analysis revealed that low category levels of vitamin D are associated with decreased sperm concentration, motility, and altered morphology, thus indicating optimal reproductive health depends upon adequate Vitamin D. The overall analysis of different studies has firm evidence for the significance of vitamin D in male fertility and points toward further investigation into the possibility of specific interventions that may improve the degree of vitamin D and its effect on fertility.

In other words, though the evidence for the role of vitamin D in male fertility is accumulating, findings are not consistent across all studies. While some studies have found a positive correlation between vitamin D levels and various sperm parameters, others have found no significant effects of supplementation on fertility outcomes. All future research should focus on elucidating the mechanisms by which Vitamin D influences male fertility, including hormonal interactions and the effects of Vitamin D on oxidative stress within the male reproductive system. This will help clinicians in the management and development of strategies for male infertility and promote reproductive health.

## 3 Vitamin D and sexual dysfunction

Sexual dysfunction is a distortion in sexual behaviour and stimulation that can occur at any of the different stages of the normal sexual cycle, such as desire, arousal, or erection, and orgasm (71). Low sexual desire or libido is a lack of sexual drive or motivation, either alone or with a partner. Libido is regulated primarily by the mesolimbic dopamine signaling (ventral tegmental area and nucleus accumbens) (72). Therefore, dopamine and other related trace amines that regulate dopamine neurotransmission are mainly responsible for regulating libido.

Erectile dysfunction (ED) is the inability to attain or maintain optimal penile erection required for sexual satisfaction. ED is a prevalent condition that affects a significant portion of the male population. Studies estimate that approximately 10%–30% of men develop some degree of erectile problem in the course of their lifetime (73). This is not just a physical illness, as it usually results in psychological disturbance, anxiety, and a decrease in the quality of life. Often, these feelings of guilt, shame, and reduced self-esteem are associated with ED in men, aggravating the condition and affecting relationships (57, 74). Thus, awareness about the causes of ED and consideration of various treatment modalities will be essential to address the sexual life and overall health of men.

Orgasm is the peak of sexual pleasure and is characterized by intense pleasurable feelings in the genitals, and it is an accompaniment to ejaculation. Orgasm is a complex physiological process that involves a cascade of physical and psychological changes, including muscle contractions, hormonal releases, and altered brain activity, leading to a peak sensation of pleasure and a release of sexual tension. However, these aforementioned sexual cycles have been shown to be influenced by vitamin D status.

Recent evidence has sought to elucidate the relationship between the level of 25-hydroxy vitamin D and erectile function, with a view to the biological plausibility of an association and the potential role of vitamin D supplementation in ED treatment (30, 75). Vitamin D is primarily obtained through sun exposure and dietary intake, playing a crucial role in various physiological processes, including maintaining bone health, immune function, and cellular growth (33). Its implications for sexual health, especially erectile function, have been increasingly realized.

Crafa et al., in their meta-analysis involving cross-sectional studies, investigated the relationship between vitamin D deficiency and ED. Indeed, from the meta-analysis, their findings indicated that a lower level of 25-hydroxyvitamin D is significantly associated with the more serious forms of ED. Interestingly, this study underlined that, even though the mean level of 25(OH)D was not significantly different between the two groups of men with and without ED, patients with vitamin D deficiency had remarkably worse erectile function scores. This finding was, moreover, pronounced in eugonadal patients and therefore likely indicates vitamin D deficiency as an additional factor worsening the severity of ED, independent of testicular function. This again focuses more on Vitamin D as a modifiable risk factor in the management of erectile dysfunction.

Similarly, Demirci et al. (64) investigated how vitamin D supplementation, when added to tadalafil, one of the most commonly prescribed medications for ED, would impact treatment outcomes. The participants consisted of “vitamin D-deficient male patients” and received 4,000 IU of vitamin D3 along with tadalafil. Although the study period was not specified, the results showed that men who received vitamin D supplementation experienced a greater improvement in their erectile function compared to those who took tadalafil only. This finding suggests that vitamin D may enhance conventional ED treatment responses, leading to improved overall outcomes in patients with concomitant vitamin D deficiency. These results point out the importance of considering vitamin D levels in the comprehensive management of erectile dysfunction.

The study of Farag et al. (65) involving 3,390 US men aged ≥20 years, revealed low levels of vitamin D in men with erectile dysfunction compared to normal subjects. Furthermore, the study of Tirabassi et al. (66) concluded that ED is associated with vitamin D and is independent of testosterone level. In the same vein, a study involving fifty patients revealed that severe ED is common in patients with low vitamin D levels (67). Additionally, a study involving a large cohort of diabetic patients found that vitamin D is inversely related to intima-media thickness and directly proportional to the IIEF-5 score and cavernous peak systolic velocity (68). This same study also identified vitamin D as a major diagnostic marker for sexual dysfunction. Another study, carried out on thirty-seven dialysis patients, revealed a negative relationship between the ASEX total score and serum vitamin D (76), indicating the importance of vitamin D in sexual function. In support of these clinical studies, findings from animal models also reported a positive correlation between vitamin D levels and sexual function. For example, the study of Zhu et al. (69) established that vitamin D improved erectile function in cavernous nerve-injured rats. Also, Fernandes-Lima et al. (70) concluded that vitamin D is an important micronutrient required for penile cytoarchitecture maintenance.

Numerous mechanisms have been hypothesized to explain the relationship between sexual dysfunction and vitamin D. One of those is the inhibitory effect of vitamin D on prolactin. Prolactin is an important hormone that increases during orgasm, and it's responsible for creating the post-orgasmic refractory period by inhibiting sexual motivation and arousal (71). Additionally, an increase in prolactin disrupts the hypothalamic-pituitary-testicular axis, which is responsible for maintaining male sexual function by inhibiting testosterone and luteinizing hormone synthesis (77, 78). Hence, the inhibitory effect of vitamin D on prolactin (79) suggests that vitamin D increases sexual motivation and arousal via a hormone-dependent mechanism.

Another important mechanism that could account for the role of vitamin D in male sexual function is its role in the monoamine neurotransmitters, since the nervous system is involved in male sexual activities. One major monoamine neurotransmitter required for male sexual function is dopamine, also known as “the addiction hormone” (80). Optimal circulatory dopamine is required for the motor activities required for sexual activities (81). Additionally, in the mesolimbic tract, dopamine stimulates various motivated behaviors, such as copulation. In the medial preoptic area, it regulates genital reflexes, copulatory patterns, and specifically, sexual motivation (82). Furthermore, dopamine stimulates penile erection by acting on the oxytocinergic neurons located in the hypothalamic paraventricular nucleus and the pro-erectile sacral parasympathetic nucleus of the spinal cord (72). Vitamin D, on the other hand, has been shown to maintain optimal levels of circulating dopamine. Pertile et al. (83) established that chronic vitamin D exposure enhanced the developing neurons' capacity to release dopamine, while Lima et al. (84) revealed the protective role of vitamin D on the dopaminergic neurons and associated it with their anti-inflammatory properties (85, 86). Additionally, a clinical study (87) revealed that vitamin D enhanced amphetamine-induced dopamine release in healthy subjects. Hence, the stimulatory effect of vitamin D on dopamine could account for its sexual performance-enhancing abilities.

Another key mechanism of action of vitamin D appears to be via Nitric oxide-mediated vascular dilation, as it stimulates NO production and release (13, 88, 89). Penis is a highly vascularized organ responsible for erection, and nitric oxide is a major mediator of penile erection via its vasodilatory effects. NO is a main secretion from the endothelial cells lining the blood vessels, and it is a neurotransmitter with vasodilatory function and therefore important for erectile function. Once released from the penile smooth muscles, NO becomes activated in the presence of sexual stimulus via the dopamine-oxytocin-NO pathway (71). Once activated, NO will act on the guanosine cyclase in the corpus cavernosum smooth muscles to produce cGMP to promote a cascade of events that will eventually lead to corpora cavernosa smooth muscle relaxation and penile erection (90, 91). Okechukwu (75) supported the fact that vitamin D is associated with erectile function mostly through its effect on endothelial health. The study stated that vitamin D increases the generation of NO, an effective vasodilator, and it is also necessary for the initiation and maintenance of an erection. A proper balance and adequate availability of NO are crucial to ensure normal blood flow to the penile tissue, a key physiological mechanism underlying erectile function. Thus, from the point of view of the relationship that vitamin D shares with the production of NO, optimization of levels may improve endothelial function and enhance erectile responses. Therefore, due to the presence of vitamin D and its receptors in the endothelial cells (92) and its stimulatory effect on NO release, it is conceivable that the interaction between vitamin D and NO plays a vital role in sexual function.

## 4 Conclusion and future perspectives

Given the growing awareness of the relationship between vitamin D and erectile health, it would seem reasonable to have healthcare providers evaluate men presenting with ED for levels of vitamin D and possibly use supplementation as part of an overall treatment plan.

However, based on promising findings, further research is needed to establish definitive cause-and-effect relationships between vitamin D levels and erectile function. Questions that remain unanswered include dosage, the duration of treatment required for significant positive effects to occur, and which populations may benefit most from supplementation. Further investigation is also needed to describe in detail the biological pathways through which vitamin D impacts erectile function and to develop an understanding of potential interactions between other factors, including lifestyle, comorbid conditions, and age.

Also, the daily supplementation threshold for maximum sexual function should be determined. It should be noted that while the tolerable upper intake level of vitamin D is 4,000 IU per day, some individuals, especially those with vitamin D deficiency or certain health conditions, may require higher doses under medical supervision.
